# Temporal Appearance of Reaction-Related Signals, Qualitative Interphase Distribution, and Theoretical Investigation During CO_2_ Capture by a DETA/DMSO/H_2_O Biphasic Absorbent

**DOI:** 10.3390/molecules31213947

**Published:** 2026-11-02

**Authors:** Zhendong Chen, Hao Chen, Junyou Zhang, Chengqi Sun, Shoujun Zhang, Banglin Deng, Yin Sun, Kun Zhang

**Affiliations:** 1 Naval Architecture and Shipping College, Guangdong Ocean University, Zhanjiang 524088, China; chenzhendong666888@163.com (Z.C.); chh602@stu.gdou.edu.cn (H.C.);; 2 Guangdong Provincial Key Laboratory of Intelligent Equipment for South China Sea Marine Ranching, Guangdong Ocean University, Zhanjiang 524088, China; 3 School of Information and Control Engineering, Liaoning Petrochemical University, Fushun 113001, China

**Keywords:** CO_2_ capture, amine-based biphasic absorbent, diethylenetriamine (DETA), time-series ^13^C NMR, phase-separated ^13^C NMR, density functional theory (DFT)

## Abstract

This study investigated the temporal appearance of CO_2_-derived signals and qualitative spectral differences between rich and lean phases in a diethylenetriamine (DETA)/dimethyl sulfoxide (DMSO)/H_2_O (DTSO) biphasic absorbent using FTIR, time-series and phase-separated ^13^C NMR, and DFT calculations. CO_2_ reacted mainly with DETA, forming carbamate-related species. The earliest detectable CO_2_-derived carbonyl signal appeared at 10 min and was tentatively assigned to a terminal mono-carbamate-related environment. Additional overlapping resonances attributable to candidate carbamate environments appeared as absorption progressed. Phase-separated ^13^C NMR showed carbonyl signals in both phases. Preliminary internal-standard estimates indicated a higher amount associated with the selected DETA-related signal in the analyzed rich-phase aliquot and a slightly higher amount associated with the selected DMSO-related signal in the lean-phase aliquot. However, these estimates do not establish a complete mass balance or quantitative partition coefficients. At the original B3LYP/SMD(H_2_O) level, terminal-site candidates had lower calculated relative Gibbs free energies than corresponding central-site candidates. Representative ion-associated structures exhibited N–H···O contacts geometrically consistent with hydrogen bonding, although the relative electronic-energy ordering of the ion-pair models was sensitive to D3(BJ) dispersion treatment. Therefore, the calculations provide a model-dependent comparison of candidate-structure stability, not definitive predictions of solution-phase species populations or ion-pair formation.

## 1. Introduction

As the scale of international trade continues to expand, the shipping industry plays an increasingly important role in global freight transportation. However, CO_2_ emissions generated during maritime transport have become a major concern regarding greenhouse gas mitigation in the maritime sector. The 2023 Strategy on Reduction of GHG Emissions from Ships adopted by the International Maritime Organization (IMO) calls for international shipping to achieve net-zero greenhouse gas emissions by or around 2050 [1]. Against this background, onboard carbon capture, utilization and storage (CCUS) technologies have attracted increasing attention. Compared with carbon capture from conventional land-based stationary sources, onboard carbon capture systems are subject to more stringent constraints associated with available space, energy consumption, and port handling conditions, thereby imposing higher requirements on the absorption capacity, regeneration energy consumption, stability, and safety of absorbents [2,3,4]. Although conventional aqueous monoethanolamine (MEA) solutions exhibit favorable CO_2_ absorption rates and benefit from a relatively mature process framework, they suffer from high regeneration energy consumption as well as corrosion, volatility, and degradation issues. These limitations can become even more pronounced in onboard carbon capture applications [5,6]. Therefore, the development of energy-efficient absorbents is of considerable importance not only for onboard carbon capture systems but also for CO_2_ capture applications in general. In recent years, biphasic/phase-change absorbents have been regarded as an effective strategy for reducing regeneration energy consumption because they can separate into a CO_2_-rich phase and a CO_2_-lean phase after CO_2_ absorption, thereby reducing the amount of solvent that must be treated during regeneration [7,8,9]. Current research has extensively investigated biphasic absorbents from the perspectives of absorbent formulation, CO_2_ absorption/desorption performance, phase-separation regulation, and reaction mechanisms. Zhang et al. pointed out that amine-based biphasic absorbents can reduce regeneration energy consumption through phase separation, although volatility and stability remain major challenges limiting their further application [10]. Luo et al. developed a biphasic absorbent composed of diethylenetriamine (DETA), sulfolane, and water, and investigated its CO_2_ absorption behavior at different temperatures and partial pressures, demonstrating favorable CO_2_ absorption performance and potential for energy-efficient capture [11]. Wang et al. regulated the phase-separation behavior of a DETA–PMDETA blended-amine system by introducing sulfolane, thereby increasing the CO_2_ loading in the rich phase and reducing the rich-phase volume, which was beneficial for lowering regeneration energy consumption [12]. In addition to evaluating absorption performance, phase-separation behavior, and regeneration energy consumption, related studies have combined NMR spectroscopy with molecular dynamics simulations or quantum chemical calculations to elucidate the distribution of absorption products, intermolecular interactions, and phase-separation mechanisms at the molecular level [13,14]. Overall, considerable progress has been made in DETA-based biphasic absorbents in terms of absorption/desorption performance, phase-separation regulation, regeneration energy consumption, and, to some extent, the mechanisms underlying phase separation. However, substantial differences in organic solvents, phase-separation agents, and water contents among different systems may lead to variations in the composition of absorption products and their intermolecular interactions. Therefore, for a specific DETA-based absorbent system, further investigation of the absorption products and molecular interactions remains necessary.

Jiang et al. screened various combinations of amine absorbents and phase-separation agents for onboard CO_2_ capture applications and ultimately identified a biphasic absorbent composed of diethylenetriamine (DETA), dimethyl sulfoxide (DMSO), and water. This absorbent exhibited relatively high CO_2_ absorption and desorption capacities accompanied by low regeneration energy consumption and was considered a low-energy phase-change absorbent with potential for onboard applications [15]. Wang et al. developed a DETA/DMSO nonaqueous solid–liquid biphasic absorbent and demonstrated, using FTIR, ^13^C NMR, TG–DSC, XRD, and DFT calculations, that CO_2_ absorption produced DETA-carbamate, protonated DETA, and a small amount of carbamic acid. The highly polar carbamate and protonated DETA species precipitated to form the solid phase, thereby contributing to a substantial reduction in regeneration energy consumption [16]. In addition, reducing the water content can alleviate the high sensible heat and heat of vaporization associated with water; however, the introduction of organic solvents may also alter the types and distribution of CO_2_ absorption products as well as their intermolecular interactions [17]. Although the favorable CO_2_ capture and regeneration performance of the DETA/DMSO/H_2_O system has been demonstrated, the temporal appearance of CO_2_-derived signals during absorption and their phase-dependent spectral characteristics after phase separation remain insufficiently understood.

The combination of spectroscopic characterization and quantum chemical calculations is an important approach for elucidating the mechanisms of amine-based absorbents. For example, Perinu et al. pointed out that NMR spectroscopy can provide direct molecular-level information on reaction species and interactions in amine–CO_2_–H_2_O systems [18]. Barzagli et al. further used ^13^C NMR spectroscopy to compare reversible CO_2_ absorption in aqueous and nonaqueous amine-based absorbents and to identify carbon-containing species such as carbamate and carbonate/bicarbonate [19]. The CO_2_–amine reaction is not a simple physical dissolution process. Instead, it involves the nucleophilic attack of the amine on CO_2_, proton transfer, and reactions involving water [20]. In DETA/DMSO systems, the formation of carbamate, protonated amine, and a small amount of carbamic acid may also be accompanied by intermolecular association [16]. Therefore, spectroscopic characterization is needed to identify the absorption species and assign the corresponding spectral peaks [18,19]. Theoretical calculations can further be used to analyze the structures, ion-pairing modes, and relative stabilities of possible products [16,20].

Although previous studies have investigated the absorption performance, phase-separation behavior, and reaction products of DETA-based biphasic absorbents using NMR spectroscopy and theoretical calculations, the temporal appearance of CO_2_-derived signals and their phase-dependent distribution in the specific DETA/DMSO/H_2_O (DTSO) system remain insufficiently understood. The present study does not aim to provide a comprehensive evaluation of the overall suitability of the DTSO absorbent for onboard application. Instead, it focuses on a specific mechanistic aspect of the previously screened DETA/DMSO/H_2_O formulation. Accordingly, time-series ^13^C NMR was employed to track the temporal appearance of reaction-related signals during CO_2_ absorption, while phase-separated ^13^C NMR was used to qualitatively compare the spectral characteristics of the rich and lean phases. FTIR spectroscopy and DFT calculations were further employed to support the interpretation and tentative assignment of major reaction-related signals and to compare the relative thermodynamic stabilities of representative candidate structures. By integrating these approaches, this work provides molecular-level insight into the temporal appearance of CO_2_-derived signals and the phase-dependent spectral characteristics of the DTSO biphasic system.

## 2. Results and Discussion

### 2.1. FTIR Characterization of Functional Group Changes Before and After CO_2_ Absorption

**Figure 1:**
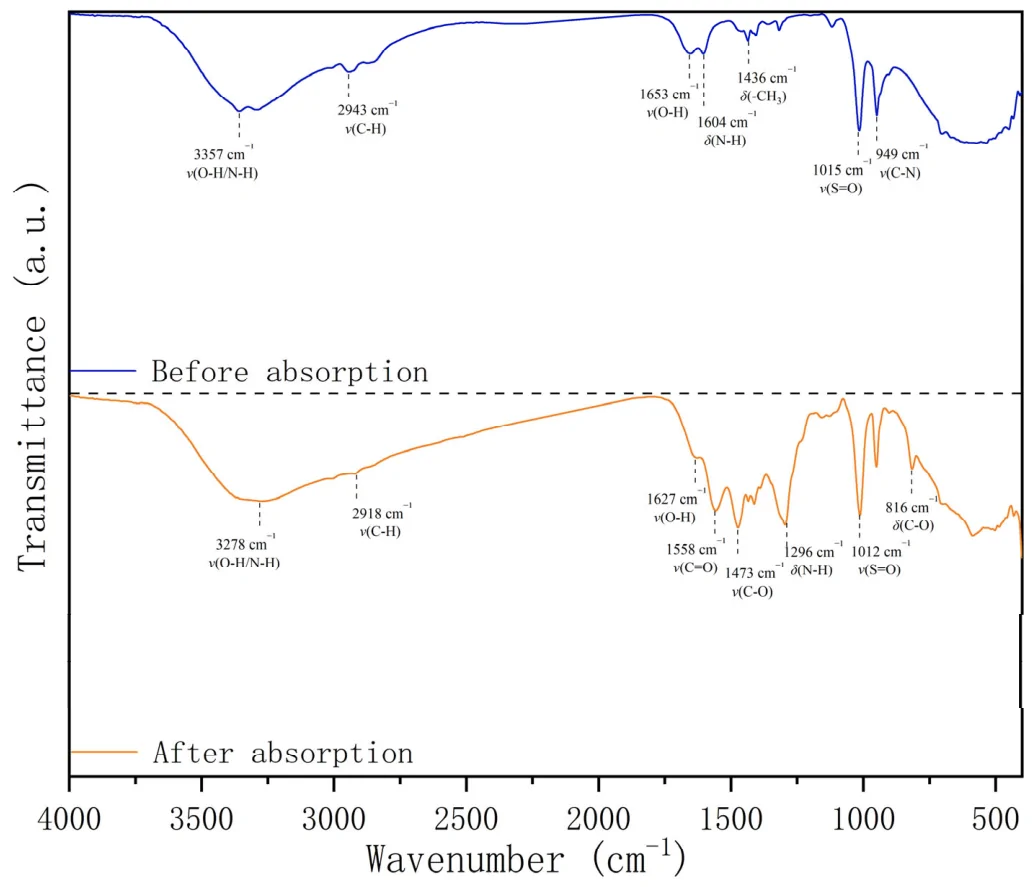
FTIR spectra of the DTSO absorbent before and after CO_2_ absorption. The horizontal dashed line serves as a visual separator between the vertically offset spectra before and after CO_2_ absorption.

Figure 1 shows the FTIR spectra of the DTSO absorbent before and after CO_2_ absorption. Before absorption, the spectrum mainly exhibited the characteristic bands of DETA, DMSO, and H_2_O. The broad band around 3357 cm^−1^ was assigned to O–H/N–H stretching vibrations, mainly associated with water molecules and the amine groups of DETA. The broadness of this band suggests the presence of pronounced hydrogen-bonding interactions in the fresh absorbent. The band around 2943 cm^−1^ was attributed to C–H stretching vibrations of DETA and DMSO. The band at approximately 1653 cm^−1^ was associated with a water-related vibrational mode, whereas that around 1604 cm^−1^ was assigned to N–H bending vibrations of the amine groups. In addition, the band around 1436 cm^−1^ was mainly attributed to methylene or alkyl bending vibrations of DETA. The strong band near 1015 cm^−1^ corresponded to the characteristic S=O stretching vibration of DMSO, and its relatively low wavenumber may be associated with hydrogen-bonding interactions between DMSO and H_2_O [21,22]. The band around 949 cm^−1^ was assigned to C–N stretching vibrations of DETA. Overall, these bands indicate that the individual components of the DTSO system retained their characteristic functional-group features before CO_2_ absorption.

After CO_2_ absorption, noticeable changes were observed in several spectral regions. The O–H/N–H stretching band shifted from approximately 3357 to 3278 cm^−1^, accompanied by a change in band shape. These changes suggest alterations in the hydrogen-bonding network and the interactions involving amine groups after CO_2_ absorption [16]. The C–H stretching band shifted from 2943 to approximately 2918 cm^−1^. The persistence of this band indicates that the C–H-containing structures of DETA and DMSO remained present after CO_2_ absorption. Compared with the water-related band near 1653 cm^−1^ before absorption, the corresponding band shifted to approximately 1627 cm^−1^ after CO_2_ absorption. This shift suggests changes in the local environment and hydrogen-bonding interactions of water molecules following CO_2_ absorption.

In the sample after CO_2_ absorption, a new absorption band appeared at approximately 1558 cm^−1^, representing one of the key spectral changes associated with the chemical binding of CO_2_. This band may be attributed mainly to the asymmetric stretching vibration of COO^−^ in carbamate species [23], suggesting that the amine groups of DETA were involved in the formation of COO^−^-containing absorption products. The new band near 1296 cm^−1^ may be associated with the C–N stretching or N–H bending vibration of carbamate species, further indicating the involvement of amine groups in the CO_2_ binding process [24]. Meanwhile, the S=O stretching band of DMSO was observed at approximately 1015 cm^−1^ in the fresh absorbent and at approximately 1012 cm^−1^ after CO_2_ absorption. Given the FTIR spectral resolution of 4 cm^−1^, this small difference was not interpreted as a definitive peak shift. The persistence of the S=O band nevertheless confirms that DMSO remained present in the system after CO_2_ absorption [22,25].

In addition to the carbamate-related features, new absorption bands were observed at approximately 1473 and 816 cm^−1^ after CO_2_ absorption. These bands may be associated with bicarbonate/carbonate-related vibrations; however, the assignments are not unique because overlapping contributions from other vibrational modes may occur in the present mixed-solvent system. Nevertheless, FTIR alone is insufficient to unambiguously confirm these inorganic carbon species [26,27].

Overall, these spectral changes support the occurrence of chemical interactions between CO_2_ and the DTSO system. This is consistent with previous studies on the DETA/DMSO system [16]. It should be noted that considerable overlap exists among the FTIR bands, particularly in the 1500–1650 cm^−1^ region, where contributions from COO^−^ vibrations, N–H bending vibrations, and the water-related vibrational modes may coexist. Therefore, FTIR alone cannot reliably distinguish DETA-carbamate, dicarbamate, and different protonated species [28]. Further ^13^C NMR analysis is therefore required to investigate the distribution of CO_2_-derived species between the rich and lean phases and their evolution with absorption time.

### 2.2. Time-Series ^13^C NMR Analysis of CO_2_-Derived Signals

**Figure 2:**
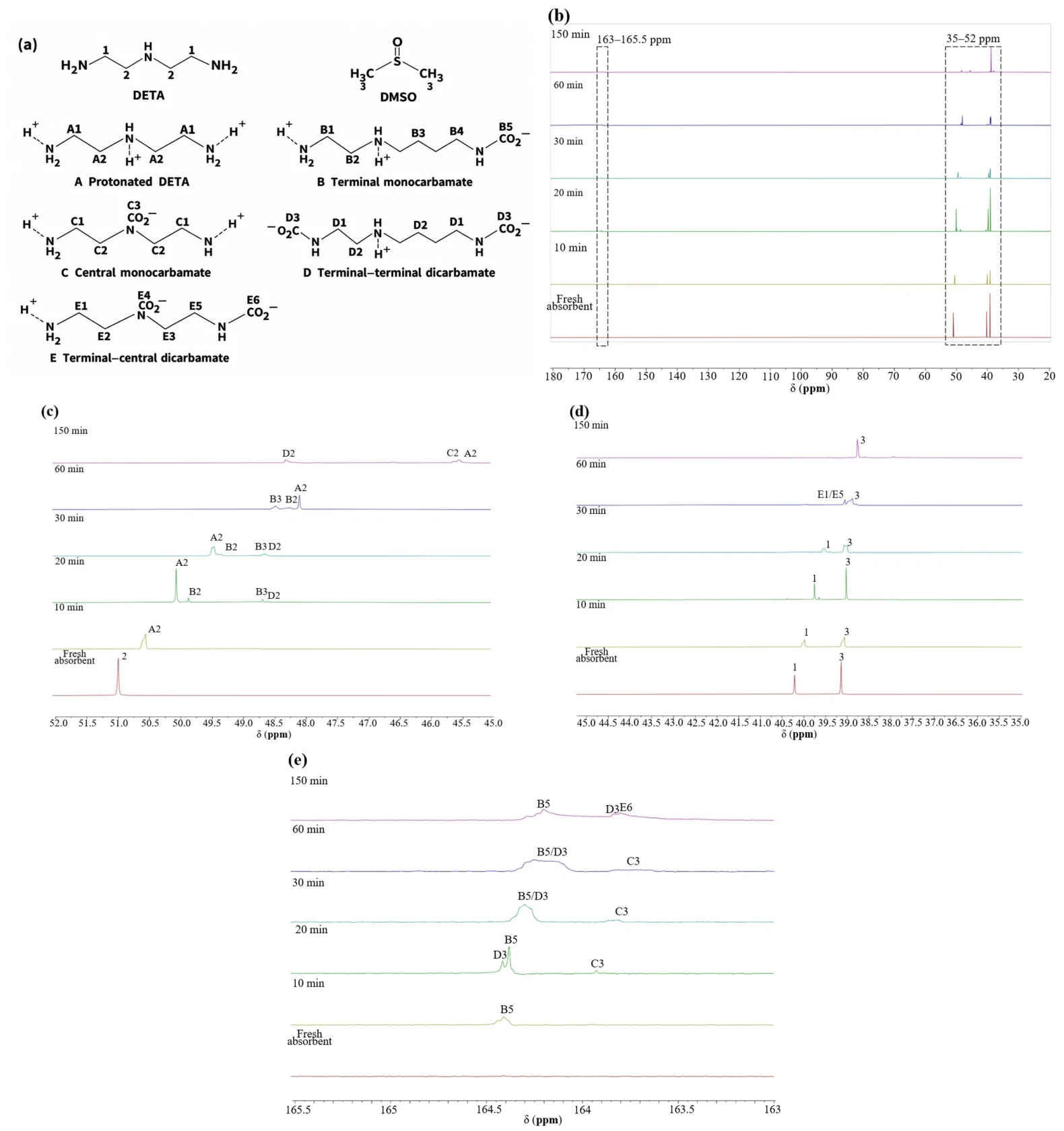
Time-series ^13^C NMR spectra of the DTSO absorbent at different CO_2_ absorption times: (**a**) structures and carbon atom numbering of candidate DETA, DMSO, and DETA–CO_2_ reaction species; (**b**) full spectra; (**c**) enlarged spectra in the 52.0–45.0 ppm region; (**d**) enlarged spectra in the 45.0–35.0 ppm region; and (**e**) enlarged spectra in the 165.5–163.0 ppm characteristic region of carbon-containing absorption products. The letters and numbers assigned to the NMR signals correspond to the candidate structures and carbon atom numbering shown in (**a**). Different colors distinguish the spectra recorded at different absorption times, as indicated alongside the spectra.

To investigate the temporal appearance and spectral changes in CO_2_-derived signals during absorption, ^13^C NMR measurements were performed on the fresh absorbent and the samples collected after 10, 20, 30, 60, and 150 min of absorption, as shown in Figure 2. The NMR sampling times and the averaged CO_2_ loadings obtained from independent replicate absorption experiments conducted under identical conditions are summarized in Table 1.

The 150 min sample represents a late-stage time-series sample rather than the final absorption endpoint.

In this study, the time-series ^13^C NMR spectra were analyzed primarily as a function of absorption time. The representative CO_2_ loadings listed in Table 1 were obtained independently from replicate absorption experiments under identical operating conditions and are provided to indicate the approximate absorption state corresponding to each sampling time. The characteristic signals in the present system were tentatively assigned by referring to the peak assignments reported for the DETA–CO_2_ system at different CO_2_ loadings in Ref. [29]. Because DMSO is present in the present system, the reported chemical shifts were used mainly to assist in candidate structural assignments rather than as the sole basis for unambiguous identification of individual signals. The full time-series spectra and enlarged spectra over different chemical-shift regions are shown in Figure 2b–e.

Relatively rapid proton exchange may occur during the reaction of DETA with CO_2_. Previous studies have shown that, in amine–CO_2_–H_2_O systems, rapid proton exchange makes it difficult to directly distinguish species such as free and protonated amines by ^13^C NMR, and the corresponding signals are often observed as time-averaged resonances [30]. Therefore, some carbon signals from unreacted DETA, protonated DETA, and different carbamate species may shift or overlap. Accordingly, this section mainly provides a qualitative analysis of the temporal appearance of CO_2_-derived signals based on the appearance of new peaks, changes in chemical shifts and peak shapes, and the order in which different signals became detectable. Because quantitative integration of the characteristic peaks was not performed, the relative abundances of different species were not inferred from peak heights. The appearance of a resonance is interpreted only as the corresponding candidate species becoming detectable under the present acquisition conditions; it does not establish its exact formation time or imply a monotonic increase in concentration.

In the spectrum of the fresh absorbent, only the characteristic signals of DETA and DMSO were observed. The signals of methylene carbons C2 and C1 in DETA appeared at 50.96 and 40.31 ppm, respectively, while the signal of methyl carbon C3 in DMSO was observed at 39.14 ppm [31]. At this stage, no obvious signals were detected in the 163.0–165.5 ppm region, indicating that no detectable resonances attributable to carbonyl-containing CO_2_-derived species were observed before CO_2_ absorption.

After 10 min of absorption, a new carbonyl carbon signal was first observed at 164.40 ppm. Based on the literature and the structural numbering shown in Figure 2a, this signal was tentatively assigned to B5, the carbonyl carbon of the terminal mono-carbamate. Meanwhile, the DETA signal originally located at 50.96 ppm shifted to 50.58 ppm, and an A2-related signal also became detectable. In the 35–45 ppm region, the main signals were observed at 40.00 and 39.08 ppm. These spectral changes are consistent with the possible appearance of a terminal mono-carbamate-related environment together with protonated DETA at the early sampling stage. These spectral changes reflect alterations in the chemical environments of the carbon atoms adjacent to the amine groups [14].

After 20 min of absorption, multiple adjacent signals appeared in the carbonyl region at 164.41, 164.37, and 163.92 ppm. The closely spaced signals near 164.4 ppm may contain overlapping contributions from B5- and D3-related environments, whereas the signal at 163.92 ppm was tentatively associated with a C3-related environment. Considering the similar chemical environments of different carbamate species, individual assignments of these signals remain tentative. Meanwhile, signals at 50.03, 49.84, 48.65, and 48.58 ppm appeared in the 45–52 ppm region and were tentatively assigned to carbon environments associated with A2-, B2-, B3-, and D2-type carbamate species, respectively. In the 35–45 ppm region, the main signals were located at 39.76 and 39.02 ppm. Compared with the 10 min sample, additional signals possibly related to the central mono-carbamate and dicarbamate species appeared at 20 min, indicating that additional carbon environments tentatively associated with candidate carbamate species became detectable as the absorption time increased.

When the absorption time was extended to 30 min, the carbonyl signal near 164.30 ppm became noticeably broadened, and a shoulder appeared at approximately 164.26 ppm, possibly due to overlap between the B5- and D3-related signals. A signal around 163.80 ppm, tentatively associated with a C3-related carbamate carbonyl environment, remained observable. In the 45–52 ppm region, the main signals further shifted to approximately 49.43, 49.31, 48.65, and 48.61 ppm and exhibited varying degrees of broadening and overlap. These spectral features are consistent with the possible coexistence of multiple DETA-related chemical environments, tentatively associated with protonated DETA and different carbamate structures.

After 60 min of absorption, two relatively broad signals were mainly observed at 164.22 and 163.73 ppm. The former may result from overlap between B5- and D3-related signals, whereas the latter may correspond to a C3/D3-related carbamate carbonyl environment. In the 45–52 ppm region, the main signals were located at 48.45, 48.22, and 48.07 ppm, indicating further changes in the chemical environments of the DETA backbone carbons, possibly related to the coexistence of CO_2_ binding at different amine sites and different protonation states [32]. In the 35–45 ppm region, a relatively weak signal was also observed near 39.05 ppm. This signal may be associated with a methylene carbon in an E-type structure; however, because it is very close to the DMSO signal, a unique assignment cannot be made at present.

After 150 min of absorption, signals near 164.20, 163.83, and 163.78 ppm were resolved in the carbonyl region. The signal at 164.20 ppm was tentatively assigned to a B5-related terminal mono-carbamate environment. The signals at 163.83 and 163.78 ppm were tentatively associated with D3- and E6-related carbamate carbonyl environments, respectively; however, contributions from overlapping carbamate species cannot be completely excluded. The persistence of the terminal mono-carbamate-related signal indicates that this environment remained detectable in the 150 min sample. Signals tentatively associated with terminal–terminal and terminal–central dicarbamate environments (D3 and E6, respectively) became detectable at the late sampling stage. In the 45–52 ppm region, the signal at 48.29 ppm may be associated with D2, whereas the overlapping signals near 45.62 and 45.53 ppm may be related to C2 and A2. However, because their chemical shifts are very close, the individual peaks are difficult to distinguish unambiguously.

Overall, the time-series ^13^C NMR spectra showed a progressive increase in the number and complexity of detectable CO_2_-related resonances with increasing absorption time. At the early sampling stage, signals tentatively associated with a terminal mono-carbamate environment and protonated DETA were observed. Additional signals tentatively associated with central mono-carbamate and dicarbamate environments became detectable at later sampling times. Because of substantial peak overlap, small chemical-shift differences, and the nonquantitative acquisition conditions, these observations describe the temporal appearance of candidate signals rather than a definitive sequence of product formation. Therefore, the present ^13^C NMR data do not by themselves establish the relative reaction rates or kinetic preference of the different amine sites in DETA [33].

In addition, the C3 signal of DMSO was observed throughout the entire absorption process, and no new signals that could be clearly assigned to products formed by direct reaction between DMSO and CO_2_ were detected. This is consistent with previous studies on DETA/DMSO systems, in which DETA acts as the main chemically reactive absorbent component while DMSO serves as an organic solvent and contributes to the formation of the phase-separation environment [16]. These results indicate that, under the present experimental conditions, DETA was the main component responsible for chemical CO_2_ capture. The DMSO peak position showed certain variations at different absorption times, which may be related to changes in solvent polarity, hydrogen-bonding environment, and sample composition during absorption. However, changes in chemical shift alone are insufficient to demonstrate that DMSO participated in a chemical reaction [34].

### 2.3. ^13^C NMR Analysis of Products in the Lean and Rich Phases

**Figure 3:**
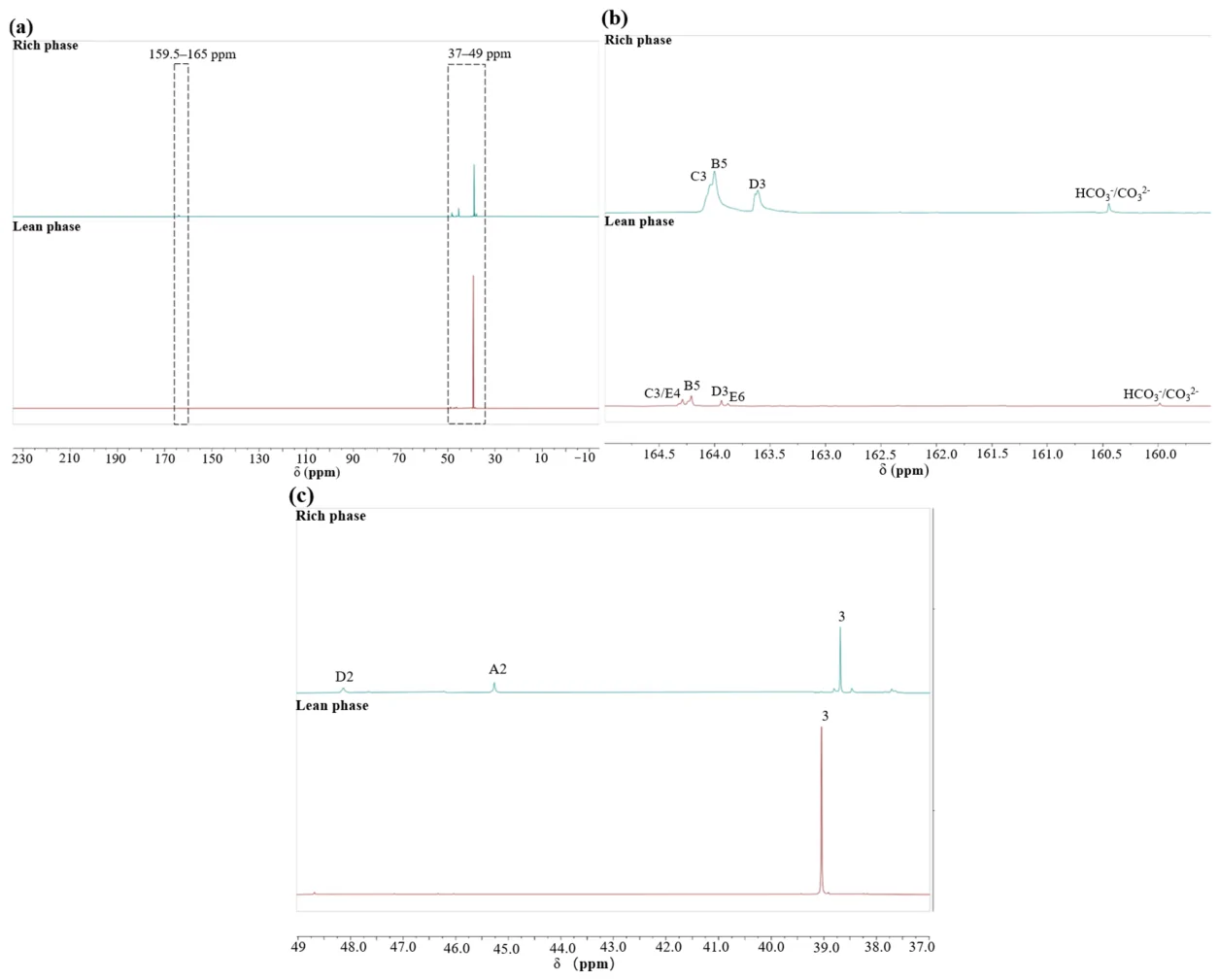
^13^C NMR spectra of the rich and lean phases of the DTSO absorbent: (**a**) full ^13^C NMR spectra of the rich and lean phases; (**b**) enlarged spectra of the 159.5–165.0 ppm region corresponding to CO_2_-derived carbon-containing species; and (**c**) enlarged spectra of the 37.0–49.0 ppm region corresponding to DETA- and DMSO-related carbon signals. The carbon signal labels correspond to the candidate structures and carbon atom numbering shown in Figure 2a.

To investigate the phase-dependent distribution of components and reaction products after CO_2_ absorption, the lean and rich phases were analyzed separately by ^13^C NMR. Unlike the time-series samples shown in Figure 2, the phase-separated samples were collected only after the predefined absorption endpoint had been reached and the absorbent had been allowed to settle sufficiently for phase separation. The full spectra of the lean and rich phases are shown in Figure 3. Enlarged spectra of the carbonyl carbon region at 159.5–165.0 ppm and the aliphatic carbon region at 37–49 ppm are also presented.

Rich-phase analysis: As shown in Figure 3b, distinct CO_2_-derived carbon signals were observed at 164.05, 164.00, and 163.61 ppm. Based on the time-series spectra and the previous peak assignments, these signals were mainly associated with C3, B5, and D3 in mono-carbamate and dicarbamate structures. This suggests that the rich phase contained signals tentatively associated with mono-carbamate and dicarbamate environments derived from DETA–CO_2_ interactions. The weak signal at 160.45 ppm may be associated with HCO_3_^−^/CO_3_^2−^ species. However, this tentative assignment does not unambiguously confirm the formation of inorganic carbonate species in the present system. Zhang et al. also observed DETA-carbamate signals in the 163–165 ppm region of the rich phase of a DETA-based biphasic absorbent. They further suggested that the CO_2_ reaction products were mainly distributed in the rich phase [14]. As shown in Figure 3c, signals assigned to D2 and A2 were observed at 48.13 and 45.26 ppm, respectively. This indicates that the rich phase contained both DETA reaction products and unreacted or differently protonated DETA species. The C3 signal at 38.69 ppm was assigned to the methyl carbon of DMSO, indicating that DMSO remained present in the rich phase after phase separation.

Lean-phase analysis: Multiple adjacent carbonyl carbon signals were still observed in the lean phase within the 164.29–163.88 ppm region. Based on the time-series spectra and the previous assignments of DETA–CO_2_ species, these signals may be associated with the candidate carbamate structures B, C, D, and E. This suggests that signals tentatively associated with DETA–CO_2_ reaction products remained detectable in the lean phase after phase separation. Zhou et al. observed HCO_3_^−^/CO_3_^2−^-related signals at approximately 160.2 ppm in a PMDETA/DETA biphasic system and found that carbamate and inorganic carbon-containing products were mainly enriched in the lower rich phase [35]. Considering the weak signals near 160.45 and 159.99 ppm in the present system, a small amount of HCO_3_^−^/CO_3_^2−^ species may be present after CO_2_ absorption. The chemical shifts in the same types of species differed to some extent between the lean and rich phases. This may be related to differences in the compositions of DMSO, H_2_O, and DETA between the two phases, as well as differences in local polarity, hydrogen-bonding interactions, and protonation environments.

In the 37–49 ppm region, the lean-phase spectrum exhibited a prominent signal at approximately 39.05 ppm, tentatively associated with the methyl carbon of DMSO, whereas fewer resolved DETA-related aliphatic signals were observed than in the rich-phase spectrum. DMSO-related and CO_2_-derived signals remained detectable in both phases. These qualitative spectral observations suggest differences in the chemical environments and component distributions of the separated phases. However, differences in unnormalized signal intensities alone are insufficient to establish the extent of component enrichment or preferential partitioning. Similar phase-dependent component distributions have been reported in other amine-based biphasic absorbent systems [36,37].

**Figure 4:**
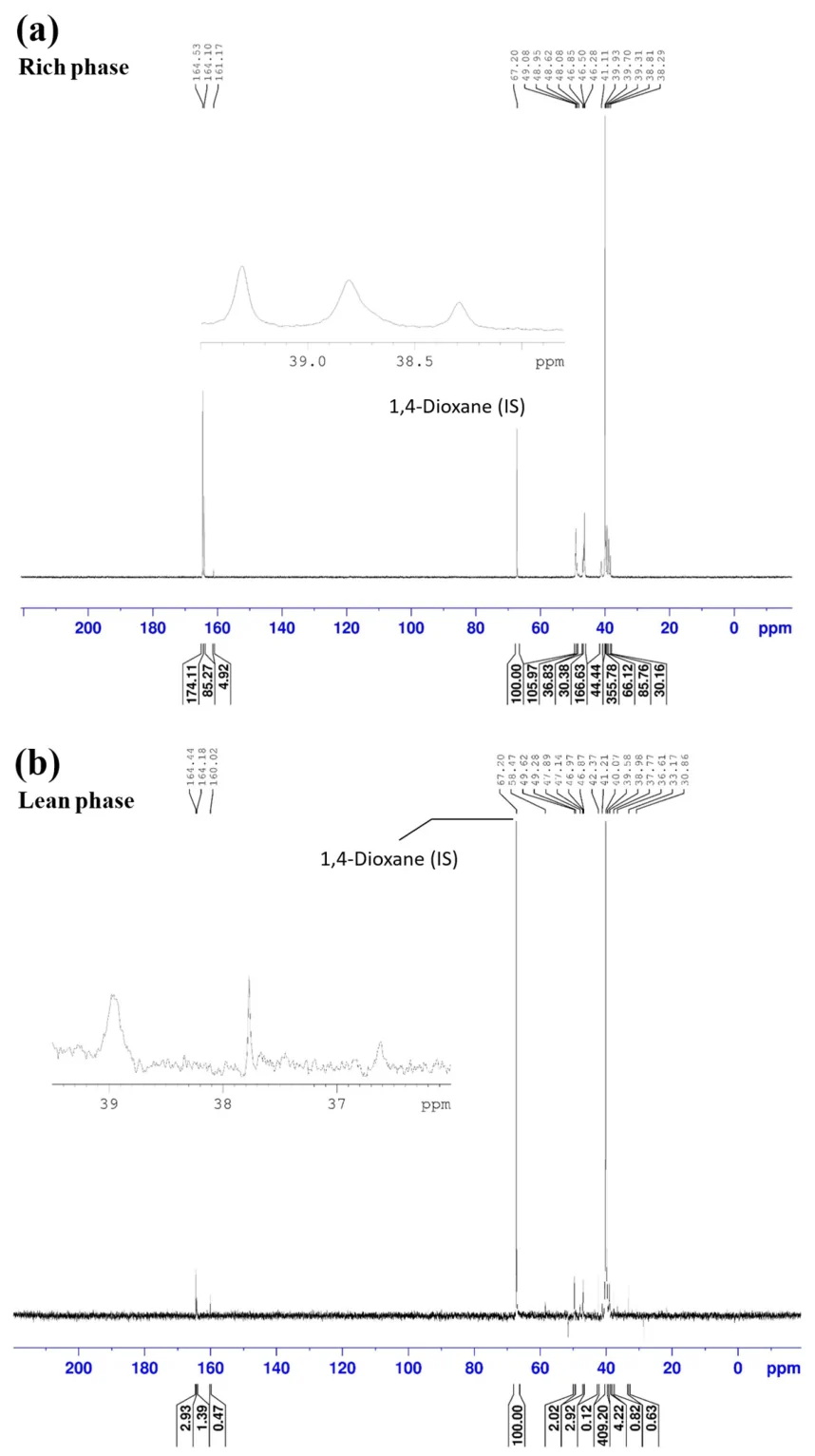
Internal-standard-assisted ^13^C NMR spectra of the phase-separated DTSO absorbent: (**a**) rich phase and (**b**) lean phase. The 1,4-dioxane signal at approximately 67.20 ppm was used as the integration reference.

To further investigate the compositional differences between the separated phases, internal-standard-assisted ^13^C NMR analysis was performed, as shown in Figure 4. The resonance of 1,4-dioxane at approximately 67.20 ppm was used as the integration reference, with its integral normalized to 100. The selection of DETA-related signals was guided by the previously reported ^13^C NMR assignments of DETA carbon environments by Ye et al. [38], although differences in solvent composition may affect the chemical shifts. For the 0.50 mL phase samples, the selected DETA-related signals were observed at approximately 38.81 ppm in the rich phase and 39.00 ppm in the lean phase. The selected DETA-related signals were tentatively assigned to a methylene-carbon environment, with each selected signal representing one carbon atom (*c_i_* = 1). The DMSO-related signal, integrated in the region centered at approximately 39.42 ppm, was treated as representing its two equivalent methyl carbons (*c_i_* = 2). Because the selected signals lie in a crowded aliphatic region, these assignments should be regarded as tentative rather than definitive structural identifications. The relative integrals of the selected DETA-related signals were 85.76 and 4.22 in the rich and lean phases, respectively. According to Equation (5), these values corresponded to estimated amounts of approximately 2.01 and 0.0989 mmol in the analyzed 0.50 mL aliquots, respectively. For DMSO, the corresponding integrals were 355.78 and 409.20, yielding estimated amounts of approximately 4.17 and 4.80 mmol in the rich and lean phases, respectively.

For the selected carbon signals, the estimated amount associated with the DETA-related signal was higher in the rich-phase aliquot than in the lean-phase aliquot, whereas the estimated amount associated with the DMSO-related signal was slightly higher in the lean-phase aliquot. Although the estimated DMSO amounts differed slightly between the two aliquots, this difference should not be interpreted as reliable evidence of preferential phase distribution under the present measurement conditions. The relaxation delay was set to 2 s, and the relevant ^13^C T_1_ relaxation times were not independently measured; potential spectral overlap could not be fully excluded. Therefore, these results should be regarded as preliminary semi-quantitative estimates. Moreover, the estimated amounts correspond to 0.50 mL aliquots of each phase and do not represent the total amounts of these components in the separated phases or establish a complete interphase mass balance.

### 2.4. DFT Analysis of the Relative Stability and Ion Pairing of CO_2_ Absorption Products

The time-series and phase-separated ^13^C NMR results suggest that terminal and central mono-carbamates, as well as dicarbamates with different binding modes, may be present after CO_2_ absorption in the DTSO system. Because some candidate species have similar chemical shifts and proton exchange may occur in the system, it is difficult to uniquely assign certain candidate structures solely from the experimental NMR signals [30]. Gibbs free-energy and quantum chemical calculations can provide complementary information for evaluating candidate species and their relative stabilities in the absorption system. However, the calculated results may be affected by the computational model, solvent environment, and parameter settings [39]. Therefore, DFT calculations were performed in this study to compare the relative Gibbs free energies of candidate CO_2_-derived species and to investigate possible ion-pair structures formed between these species and protonated DETA. The structures discussed here are representative local minima obtained for the corresponding reaction sites and protonation modes. It should be noted that no transition-state searches or kinetic calculations were performed in this study. Therefore, the calculated relative Gibbs free energies were used only to compare the relative thermodynamic stabilities of the selected candidate structures and cannot be used to determine kinetic preferences among reaction pathways, reaction rates, or the actual order of species formation. Calculated ^13^C NMR chemical shifts were not obtained for the B, C, D, and E candidate structures. Therefore, the experimental signal assignments remain tentative and literature-guided rather than computationally validated.

**Figure 5:**
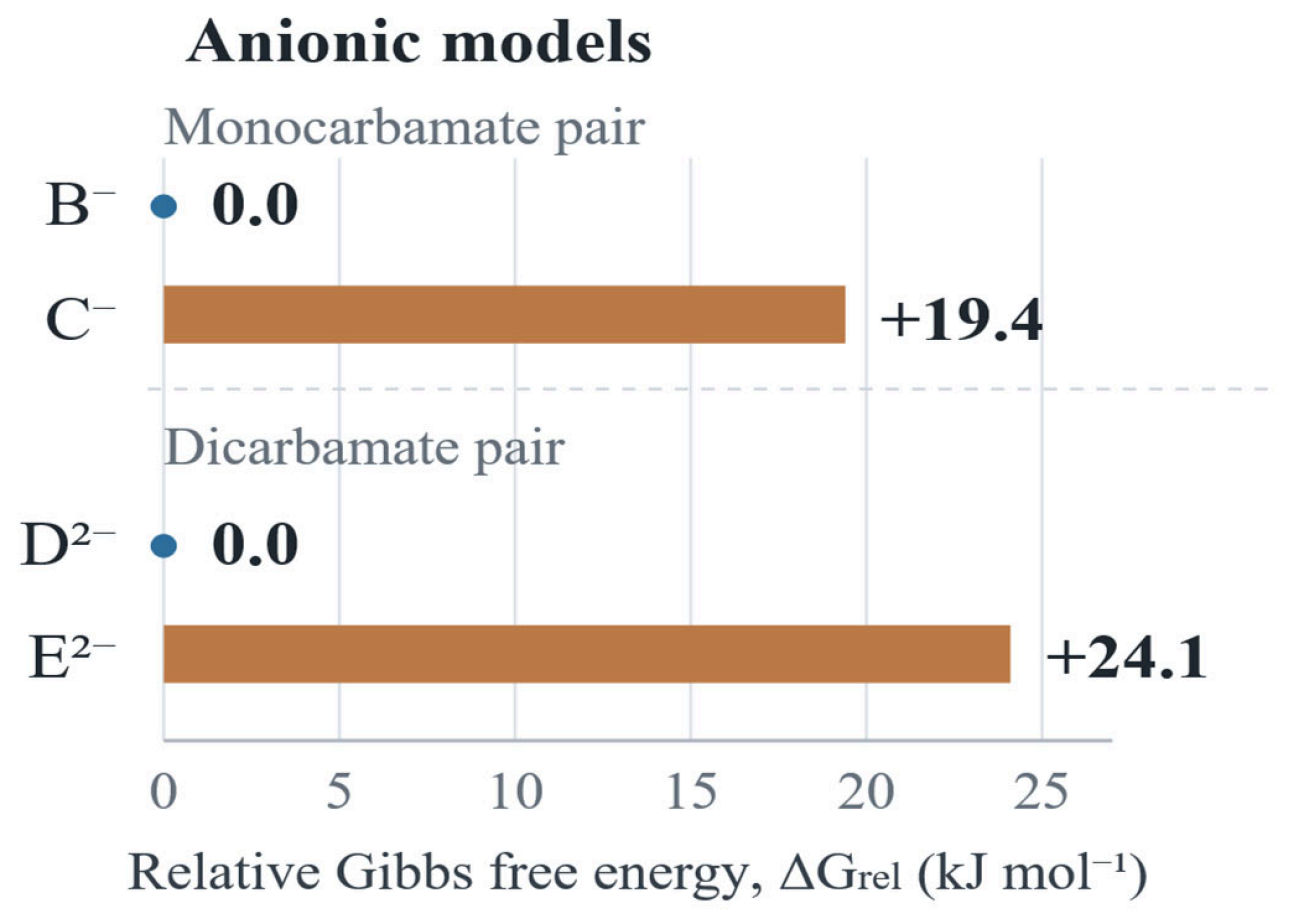
Relative Gibbs free energies of the isolated mono- and dicarbamate anion models calculated at the B3LYP/6-311++G(d,p)/SMD(H_2_O) level. Each structural pair is independently referenced to its lower-energy model (0.0 kJ mol^−1^); no cross-pair comparison is intended. The structural labels B, C, D, and E correspond to the candidate structures illustrated in Figure 2a.

As shown in Figure 5, for the mono-carbamate anion models, B^−^ formed through reaction at a terminal primary amine site was 19.4 kJ mol^−1^ lower in Gibbs free energy than C^−^ formed through reaction at the central secondary amine site. For the dicarbamate dianion models, the terminal–terminal product D^2−^ was 24.1 kJ mol^−1^ lower in Gibbs free energy than the terminal–central product E^2−^. These results indicate a relative thermodynamic preference for terminal-site carbamate candidate structures over their central-site counterparts within the selected computational models. This theoretical comparison provides complementary molecular-level information on the relative stability of plausible CO_2_-derived structures, without establishing their definitive NMR assignments or kinetic formation sequence.

**Figure 6:**
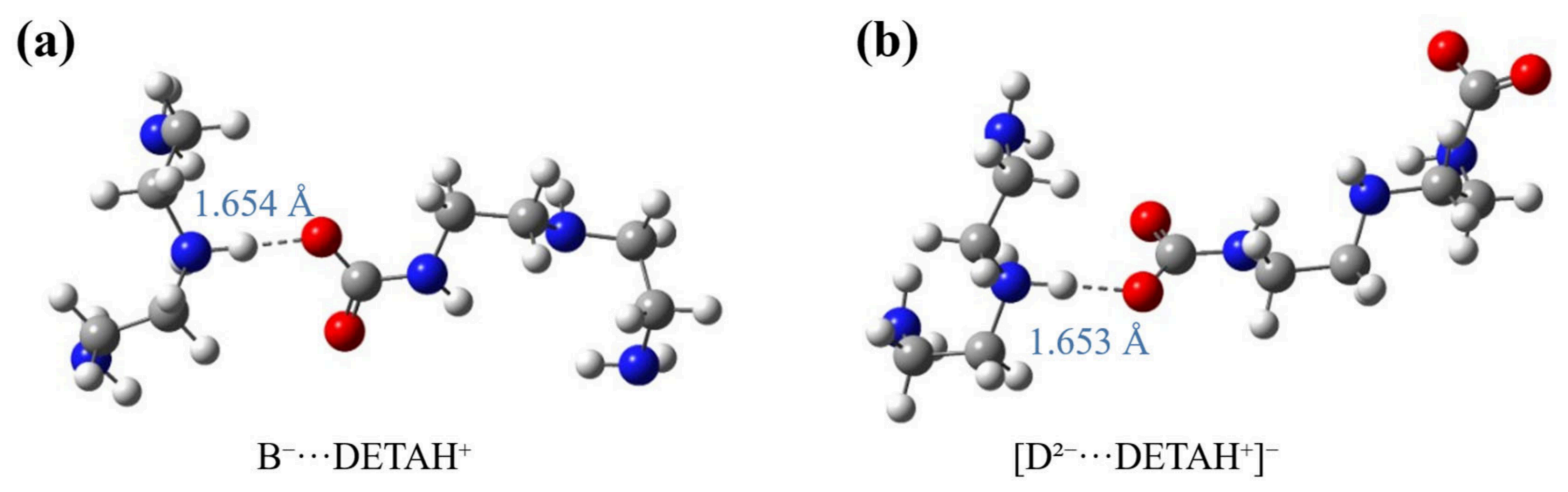
Representative optimized structures of ion-pair models: (**a**) B^−^···DETAH^+^; (**b**) [D^2−^···DETAH^+^]^−^. Gray, white, blue, and red spheres represent carbon, hydrogen, nitrogen, and oxygen atoms, respectively.

Among the two DETAH^+^ models examined, the structure protonated at the central nitrogen was approximately 2.7 kJ mol^−1^ lower in Gibbs free energy than that protonated at a terminal nitrogen. Therefore, the centrally protonated DETAH^+^ structure was used consistently in the ion-pair calculations. Carbamate formation is accompanied by proton transfer, and the resulting anions may further form ion-pair structures with DETAH^+^ [40]. The relative Gibbs free-energy differences calculated for the ion-pair models at the original B3LYP/SMD(H_2_O) level should not be interpreted as evidence of a robust energetic preference, given the sensitivity of their relative electronic energies to D3(BJ) dispersion correction. Representative optimized structures are shown in Figure 6. In these structures, the N–H groups of DETAH^+^ are oriented toward the O atoms of the carbamate groups, with H···O distances of 1.654 and 1.653 Å, respectively. These distances are geometrically consistent with N–H···O hydrogen bonding, although they do not quantify hydrogen-bond strength. It should be noted that [D^2−^···DETAH^+^]^−^ represents only a local association mode between D^2−^ and one DETAH^+^ and does not represent the complete composition of an electroneutral aggregate in solution. To assess the sensitivity of the energetic ordering to the continuum solvent model, additional single-point calculations were performed using SMD(H_2_O) and SMD(DMSO), and the results are summarized in Table S1. Although the magnitudes of the relative electronic-energy differences varied between the two continuum solvent models, the lower-energy structure within each corresponding structural pair remained unchanged. This indicates that the qualitative energetic ordering is consistent between the two continuum solvent models examined.

Additionally, dispersion-corrected single-point calculations using B3LYP-D3(BJ) were performed to evaluate the influence of dispersion interactions on the relative electronic-energy ordering, as summarized in Table S6. After inclusion of the D3(BJ) dispersion correction, the B^−^ and D^2−^ anionic structures remained lower in electronic energy than their corresponding C^−^ and E^2−^ counterparts. However, the relative energy difference between the B- and C-type ion-pair structures became very small, with a slight reversal in their energetic ordering. These results indicate that the relative energetic ordering of the ion-pair structures, particularly the B/C pair, is sensitive to dispersion treatment.

Nevertheless, neither single-solvent continuum model fully represents the specific solvation and local hydrogen-bonding environment of the actual DTSO mixture. Therefore, these additional calculations are interpreted as sensitivity analyses of the relative electronic-energy ordering to the continuum solvent model and dispersion correction, rather than definitive predictions of product selectivity in the actual mixed-solvent system.

Overall, among the isolated anionic models examined, B^−^ and D^2−^ exhibited lower relative Gibbs free energies than their corresponding C^−^ and E^2−^ structures at the original B3LYP/SMD(H_2_O) level. However, these results do not exclude the presence of C- and E-type species, because local solvation, hydrogen-bonding environments, proton exchange, and CO_2_ loading may all influence the actual species distribution. Because exhaustive conformational searches, transition-state calculations, and kinetic analyses were not performed in this study, the present results are used only to assist in evaluating the relative stabilities of candidate products and their possible ion-pairing modes. They cannot be used to determine reaction barriers, reaction rates, or the quantitative proportions of individual species.

## 3. Materials and Methods

### 3.1. Materials

Diethylenetriamine (DETA, purity ≥ 99%, CAS No. 111-40-0) and dimethyl sulfoxide (DMSO, purity ≥ 99%, CAS No. 67-68-5) were purchased from Shanghai Macklin Biochemical Co., Ltd. (Shanghai, China). CO_2_ (≥99.99 vol%) and N_2_ (≥99.99 vol%) were supplied by Zhanjiang Shiji Shuanglong Industrial Gases Co., Ltd. (Zhanjiang, China). All amine solutions were prepared using deionized water.

### 3.2. Experimental Procedure

**Figure 7:**
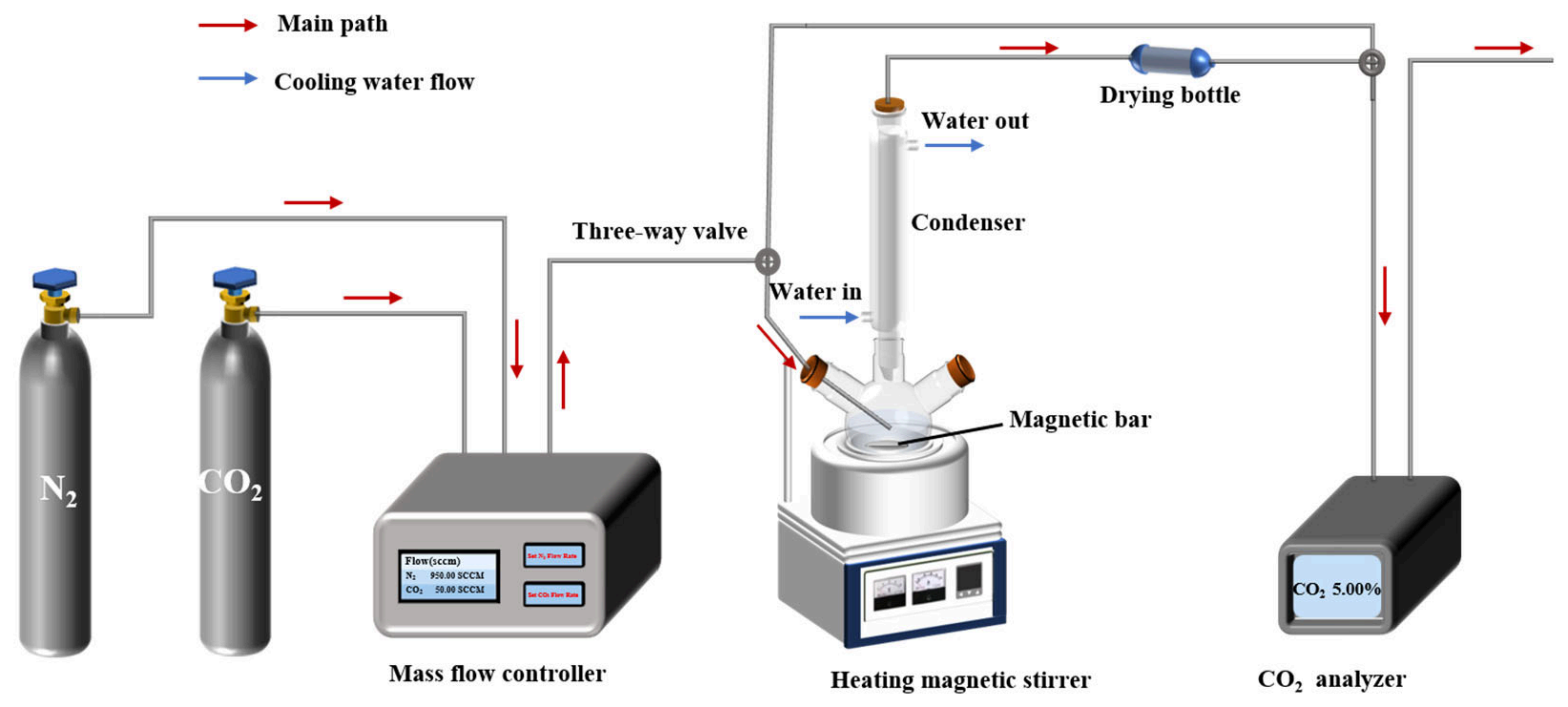
Schematic diagram of the CO_2_ absorption experimental setup.

The experimental setup for CO_2_ absorption is shown in Figure 7. The absorption experiments were conducted at 313.15 K and atmospheric pressure. The apparatus mainly consisted of seven components: gas cylinders, a gas mixing and flow control unit, a 250 mL three-necked flask, a heating magnetic stirrer, a condenser, a drying bottle, and a CO_2_ analyzer. The gas mixing and flow control unit was used to regulate the flow rates of N_2_ and CO_2_ and mix the two gases at a predetermined ratio to obtain a simulated ship exhaust gas containing 5.0 vol% CO_2_ and 95.0 vol% N_2_. The three-necked flask served as the gas–liquid absorption reactor, while the heating magnetic stirrer was used for heating and agitation. The condenser was used to cool the outlet gas and minimize absorbent loss through volatilization, and the drying bottle was used to remove moisture from the gas stream. The CO_2_ concentration in the outlet gas was continuously monitored using a PG-350 gas analyzer (HORIBA, Kyoto, Japan; CO_2_ measuring range: 0–30 vol%; linearity: ±2.0% F.S.).

Before the experiment, DETA, DMSO, and H_2_O were mixed at a mass ratio of 3:3:4. This composition was adopted based on previous formulation investigations, which considered CO_2_ absorption performance, phase-separation behavior, and rich-phase viscosity. The present study employed this composition as a fixed formulation to investigate its molecular-level absorption behavior. A total of 50.0 g of absorbent was used for each absorption experiment. The prepared absorbent was then transferred into a three-necked flask, which was placed on the magnetic stirrer with heating. The absorption temperature was maintained at 313.15 K, and the stirring speed was fixed at 1000 rpm throughout the absorption experiments. Before CO_2_ was introduced, the main gas line was purged with high-purity N_2_ to remove residual air and CO_2_ from the apparatus. The absorption experiment was initiated when the CO_2_ concentration measured by the CO_2_ analyzer reached zero.

A binary CO_2_/N_2_ gas mixture was used to simulate ship exhaust gas. The CO_2_ concentration was set at 5.0 vol% [41], with N_2_ used as the balance gas. The total gas flow rate was maintained at 1000 mL min^−1^, corresponding to CO_2_ and N_2_ flow rates of 50 and 950 mL min^−1^, respectively, with the gas flow rates controlled by D07-19B mass flow controllers (Sevenstar, Beijing, China; full-scale ranges: 400 mL min^−1^ for CO_2_ and 1000 mL min^−1^ for N_2_; accuracy: ±1.0% F.S.; factory-calibrated by the manufacturer). Before absorption, the mixed gas was first introduced through the bypass line. After the CO_2_ concentration stabilized at the preset value, the gas flow was switched to the main reaction line and bubbled through the absorbent to promote gas–liquid contact and CO_2_ absorption. The outlet gas then passed sequentially through the condenser and drying bottle before entering the CO_2_ analyzer. The outlet CO_2_ concentration was continuously recorded as a function of absorption time.

CO_2_ loading was defined as the amount of CO_2_ absorbed per mole of DETA (mol CO_2_/mol DETA). The outlet CO_2_ concentration was continuously monitored by the CO_2_ analyzer, and the gas flow rates were converted to molar flow rates using a standard molar volume of 22.414 L mol^−1^ at 0 °C and 1 atm. Assuming that N_2_ was not absorbed by the liquid phase, the outlet CO_2_ molar flow rate was calculated from the measured outlet CO_2_ mole fraction according to

(1) $$q_{\mathrm{out}}\left(t_{i}\right)=q_{N_{2}}\frac{y_{{\mathrm{CO}}_{2},\mathrm{out}}(t_{i})}{1-y_{{\mathrm{CO}}_{2},\mathrm{out}}(t_{i})}$$

where $q_{N_{2}}$ is the N_2_ molar flow rate (mol s^−1^), and $y_{{\mathrm{CO}}_{2},\mathrm{out}}(t_{i})$ is the dimensionless CO_2_ mole fraction in the outlet gas at time *t_i_*, obtained by converting the analyzer reading from vol% to a fraction.

The CO_2_ loading was calculated from the cumulative difference between the inlet and outlet CO_2_ molar flow rates:

(2) $$\alpha \left(t\right)=\frac{1}{n_{\mathrm{DETA}}}\sum_{i=0}^{N\left(t\right)}\left[q_{\mathrm{in}}-q_{\mathrm{out}}\left(t_{i}\right)\right]∆t$$

where *α*(*t*) is the CO_2_ loading at time *t*(mol CO_2_/mol DETA); *q_in_* and *q_out_* (*t_i_*) are the inlet and outlet CO_2_ molar flow rates, respectively (mol s^−1^); *n_DETA_* is the amount of DETA in the absorbent (mol); and ∆*t* is the time interval between two consecutive data points (s).

For each experiment, 15.0 g of DETA was used.

The repeatability of the CO_2_-loading results was evaluated from three independent absorption experiments conducted under identical conditions, and the values are reported as mean ± standard deviation (SD, *n* = 3). The combined standard uncertainty of the calculated CO_2_ loading was evaluated using the first-order law of propagation of uncertainty. Assuming that the input quantities were uncorrelated, for a CO_2_ loading expressed as *α* = *f*(*x*_1_,*x*_2_,…,*x_n_*), the combined standard uncertainty was calculated as

(3) $$u_{c}\left(\alpha \right)=\sqrt{\sum_{i=1}^{n}{\left(\frac{\partial \alpha }{\partial x_{i}}\right)}^{2}u^{2}}\left(x_{i}\right)$$

where *x_i_* represents an input quantity involved in the CO_2_-loading calculation and *u*(*x_i_*) is its corresponding standard uncertainty. For manufacturer-specified limits for which no probability distribution was provided, a rectangular distribution was assumed for the Type B evaluation, and the corresponding standard uncertainty was estimated as

(4) $$u_{B}=\frac{∆}{\sqrt{3}}$$

where ∆ is the half-width of the specified uncertainty interval. Detailed input uncertainties and propagated uncertainty results are provided in the Supporting Information.

During CO_2_ absorption, 1.5 mL aliquots were withdrawn from the continuously stirred bulk absorbent at selected sampling times (Fresh, 10, 20, 30, 60, and 150 min) for time-series ^13^C NMR analysis. These sampling points were selected to characterize representative stages of the absorption process. Notably, the 150 min sample was collected before the absorption endpoint was reached and therefore did not represent the final absorption state. In a separate absorption experiment conducted under the same operating conditions, CO_2_ absorption was continued until the absorption endpoint was reached. The absorbent was then allowed to settle until clear macroscopic phase separation occurred, after which the lean and rich phases were collected separately for phase-resolved ^13^C NMR analysis. Each sample was sealed immediately after collection to minimize changes in sample composition caused by exposure to air and ambient moisture.

### 3.3. Characterization and Theoretical Calculations

The DTSO samples before and after CO_2_ absorption were characterized by ^13^C NMR and FTIR spectroscopy. For ^13^C NMR analysis, samples collected at different absorption times were transferred into NMR tubes, and D_2_O was added for deuterium locking. The spectra were then recorded using a 400 MHz NMR spectrometer (Bruker, Ettlingen, Germany). For the internal-standard-assisted ^13^C NMR analysis of the rich and lean phases, 0.50 mL of each phase sample was mixed with 0.05 mL of 1,4-dioxane as an internal standard and 0.05 mL of D_2_O. The spectra were acquired using an inverse-gated decoupling pulse sequence (zgig30), with 512 scans and a relaxation delay of 2 s. The resonance of 1,4-dioxane at approximately 67.20 ppm was used as the integration reference, with its integral normalized to 100.

The amount of each component was estimated using the internal-standard integration method according to the following equation [30]:

(5) $$n_{i}=4\frac{A_{S_{n},i}n_{\mathrm{IS}}}{c_{i}A_{\mathrm{IS}}}$$

where *n_i_* is the estimated amount of component *i* (mmol); $A_{S_{n},i}$ is the integrated area of the selected signal of component *i*; *n_IS_* is the amount of 1,4-dioxane added (0.586 mmol); *c_i_* is the number of equivalent carbon atoms contributing to the selected signal; and *A_IS_* is the integrated area of the 1,4-dioxane signal, normalized to 100. The factor of 4 represents the four equivalent carbon atoms of 1,4-dioxane.

In the present calculations, *c_i_* was set to 1 for the selected DETA-related methylene-carbon signal and to 2 for the DMSO-related signal, accounting for the two equivalent methyl carbons of DMSO.

The integrated signals were used for a semi-quantitative comparison of selected DETA- and DMSO-related carbon environments in the two phases.

For FTIR analysis, the fresh absorbent and the CO_2_-loaded sample were characterized using a Fourier transform infrared spectrometer (Nicolet iS5, Thermo Scientific, Waltham, MA, USA). Liquid samples were analyzed using a liquid cell in transmittance mode over the range of 4000–400 cm^−1^, with a resolution of 4 cm^−1^ and 32 scans. After baseline correction, the spectra were used to analyze changes in characteristic absorption bands associated with different functional groups.

To assist in evaluating the stability of the CO_2_ absorption products, quantum chemical calculations were performed using Gaussian 16. Density functional theory (DFT) calculations were carried out using the B3LYP functional with the 6-311++G(d,p) basis set. Geometry optimizations and frequency calculations were performed for DETA, the DETA-carbamate anion, the protonated DETA cation, and DETA-carbamate/protonated DETA ion-pair models. The thermal corrections and Gibbs free energies were evaluated at 298.15 K and 1 atm. Although the absorption experiments were conducted at 313.15 K, the calculated Gibbs free energies were used only for qualitative comparison of the relative thermodynamic stabilities of the selected candidate structures. The SMD implicit solvation model was employed with water as the continuum solvent to approximately represent the aqueous polar environment of the DTSO system [42,43]. Because DMSO constitutes a substantial fraction of the experimental absorbent, additional single-point calculations were performed on the optimized structures using SMD(DMSO) at the same B3LYP/6-311++G(d,p) level to evaluate the sensitivity of the relative energetic ordering to the continuum solvent environment. These additional calculations were used only as a solvent-model sensitivity test of the relative electronic-energy ordering and were not intended to reproduce the complete local solvation environment of the actual DETA/DMSO/H_2_O mixture. To assess the sensitivity of the energetic ordering to dispersion treatment, additional B3LYP-D3(BJ)/6-311++G(d,p) single-point calculations with SMD(H_2_O) were performed on the geometries optimized at the original B3LYP/6-311++G(d,p)/SMD(H_2_O) level. These calculations were used only to examine the sensitivity of the relative electronic-energy ordering to dispersion treatment. The relative Gibbs free energies discussed in the main thermodynamic analysis were obtained consistently from the SMD(H_2_O) geometry optimizations and frequency calculations. All relative Gibbs free energies were compared at the same level of theory and using the same solvation model. The optimized structures were confirmed as local minima by frequency calculations, and GaussView was used for structural visualization.

## 4. Conclusions

This study employed FTIR, time-series and phase-separated ^13^C NMR, together with DFT calculations, to investigate the temporal appearance of CO_2_-derived signals during absorption and the qualitative spectral characteristics of the separated phases in the DTSO system. The FTIR results showed pronounced changes in the vibrational features associated with O–H/N–H, C–N, and COO^−^ groups after CO_2_ absorption, suggesting that DETA was involved in the formation of carbamate-related species. Time-series ^13^C NMR showed that the earliest detectable CO_2_-derived carbonyl resonance was tentatively assigned to a terminal mono-carbamate environment. Additional resonances tentatively associated with central mono-carbamate and dicarbamate environments became detectable at later sampling times. Because the spectra were not acquired under quantitative conditions, these observations describe the temporal appearance of signals rather than concentration trajectories or exact formation times. No new signals that could be unambiguously assigned to products formed by direct reaction between DMSO and CO_2_ were observed throughout the absorption process, indicating that DETA was the primary component responsible for chemical CO_2_ binding under the present conditions.

Phase-separated ^13^C NMR revealed qualitative spectral differences between the rich and lean phases, consistent with possible differences in component distribution. Nevertheless, characteristic component-related signals remained detectable in both phases, and the present data do not establish quantitative interphase partitioning.

The DFT calculations indicated that, within the original B3LYP/SMD(H_2_O) model, the terminal-site candidate structures B^−^ and D^2−^ had lower calculated relative Gibbs free energies than the corresponding central-site candidates C^−^ and E^2−^. However, D3(BJ)-corrected single-point calculations showed that the relative electronic-energy ordering of the ion-pair models was sensitive to dispersion treatment. The N–H···O contacts in representative optimized structures were geometrically consistent with hydrogen bonding but did not establish ion-pair formation in the experimental solution. Overall, this study characterizes the temporal appearance of CO_2_-derived signals, qualitative spectral differences between the separated phases, and the model-dependent relative stability of selected candidate structures. The available results do not establish quantitative interphase partition coefficients or definitive solution-phase ion-pair populations.

## Figures and Tables

**Table 1:** NMR sampling times and the averaged CO_2_ loadings obtained from independent replicate absorption experiments under identical conditions.

Sample	Absorption Time (min)	Representative CO_2_ Loading (mol CO_2_/mol DETA, Mean ± SD, n = 3)
Fresh	0	0
S1	10	0.134 ± 0.003
S2	20	0.259 ± 0.006
S3	30	0.377 ± 0.008
S4	60	0.692 ± 0.013
S5	150	1.236 ± 0.015

Note: The reported CO_2_ loadings are mean values obtained from three independent absorption experiments conducted under identical operating conditions (n = 3). They are provided as representative loadings at the corresponding NMR sampling times and are not direct sample-specific measurements of the NMR aliquots. The relative combined standard uncertainty of the calculated CO_2_ loadings ranged from 9.3% to 15.5%, mainly due to the uncertainty associated with the PG-350 CO_2_ analyzer. This uncertainty is substantially larger than the relative run-to-run SD (approximately 1.2–2.3%), indicating that instrumental uncertainty is a major contributor to the overall uncertainty. Detailed calculations are provided in the Supporting Information.

## Data Availability

The data presented in this study are available from the corresponding author upon reasonable request.

## Supplementary Materials

The following supporting information can be downloaded at: https://www.mdpi.com/article/10.3390/molecules31213947/s1, Table S1: Sensitivity of the relative electronic-energy ordering of candidate structures to the continuum solvent model; Table S2: Input quantities and standard uncertainties used for uncertainty propagation; Table S3: Propagated standard uncertainties of CO_2_ loading at different absorption times; Table S4: Individual CO_2_ loadings obtained from three independent absorption experiments; Table S5: Tentative assignment of characteristic ^13^C NMR signals in time-series and phase-separated DETA/DMSO/H_2_O samples; Table S6: Effect of D3(BJ) dispersion correction on the relative electronic energies of candidate carbamate structures and ion-pair models; Cartesian coordinates of DFT-optimized structures.
