# Supplementary material for: Temporal Appearance of Reaction-Related Signals, Qualitative Interphase Distribution, and Theoretical Investigation During CO2 Capture by a DETA/DMSO/H2O Biphasic Absorbent
Source: Molecules. 2026 Nov 2;31(21):3947. doi: 10.3390/molecules31213947 (PMC13765518; doi:10.3390/molecules31213947)
Supplement: Supplementary file 1 [file molecules-31-03947-s001.zip › molecules-4608576-supplementary.pdf]

Table S1. Sensitivity of the relative electronic-energy ordering of candidate structures to the continuum solvent model.

Structural pair	ΔE_{rel} in SMD(H_2O) (kJ mol^{-1})	ΔE_{rel} in SMD(DMSO) (kJ mol^{-1})	Lower-energy structure (both models)
B^- / C^-	20.26	18.89	B^-
D^{2-} / E^{2-}	19.16	27.53	D^{2-}
$B^-\cdots DETAH^+$ / $C^-\cdots DETAH^+$	14.19	5.49	B-type
$[D^{2-}\cdots DETAH^+]^-$ / $[E^{2-}\cdots DETAH^+]^-$	18.61	22.60	D-type

Note: For each structural pair, ΔE_{rel} = E(second-listed structure) − E(first-listed structure). Positive values therefore indicate that the first-listed structure is lower in electronic energy. The values were obtained at the B3LYP/6-311++G(d,p) level using SMD(H_2O) or SMD(DMSO). The SMD(DMSO) calculations were single-point calculations on the corresponding optimized structures and are used only to evaluate solvent-model sensitivity of the electronic-energy ordering; they should not be interpreted as Gibbs free-energy differences.

Table S2. Input quantities and standard uncertainties used for uncertainty propagation.

Input quantity	Experimental value / range	Specification used	Distribution	Standard uncertainty
CO_2 flow rate, QCO_2	50 mL min^{-1}	D07-19B; F.S. = 400 mL min^{-1}; ±1.0% F.S. = ±4 mL min^{-1}	Rectangular	2.309 mL min^{-1}
N_2 flow rate, QN_2	950 mL min^{-1}	D07-19B; F.S. = 1000 mL min^{-1}; ±1.0% F.S. = ±10 mL min^{-1}	Rectangular	5.774 mL min^{-1}
Outlet CO_2 mole fraction, y	PG-350 range: 0–30 vol%	Linearity ±2.0% F.S. = ±0.60 vol%	Rectangular	0.3464 vol% (= 0.003464 as mole fraction)
DETA mass, m_{DETA}	15.0 g	Balance resolution d = 0.01 g	Rectangular quantization interval	0.002887 g

Note: The sampling interval was 3 s. The standard molar volume V_m= 22.414 L mol^{-1} and the molar mass of DETA were treated as fixed conversion constants and were not included as independent uncertainty sources.

Table S3. Propagated standard uncertainties of CO_2 loading at different absorption times.

Absorption time (min)	Mean CO_2 loading (mol CO_2/mol DETA)	CO_2 MFC contribution	N_2 MFC contribution	PG-350 contribution	Weighing contribution	Combined uc(α)	Relative uc (%)
10	0.1344	0.007122	0.000120	0.010287	2.59×10^{-5}	0.012512	9.31
20	0.2588	0.014209	0.000297	0.020588	4.98×10^{-5}	0.025017	9.67
30	0.3771	0.021295	0.000510	0.030931	7.26×10^{-5}	0.037556	9.96
60	0.6919	0.042555	0.001394	0.062243	1.33×10^{-4}	0.075413	10.90
150	1.2355	0.106335	0.006483	0.159026	2.38×10^{-4}	0.191412	15.49

The relative combined standard uncertainty increased from approximately 9.3% at 10 min to 15.5% at 150 min. The PG-350 CO_2 concentration measurement represented the largest contribution to the propagated uncertainty, followed by the CO_2 mass flow controller, whereas the contributions from the N_2 mass flow controller and DETA weighing were comparatively small. The propagated uncertainties quantify the effects of instrumental and input-quantity specifications on the calculated CO_2 loading and should be distinguished from the experimental standard deviations obtained from the three independent absorption experiments.

Table S4. Individual CO_2 loadings obtained from three independent absorption experiments.

Time (min)	Run 1	Run 2	Run 3	Mean	SD	RSD (%)
10	0.137355	0.132566	0.133303	0.134408	0.002578	1.92
20	0.265315	0.254068	0.256907	0.258763	0.005849	2.26
30	0.386190	0.370651	0.374451	0.377097	0.008101	2.15
60	0.706966	0.684406	0.684423	0.691932	0.013020	1.88
150	1.250251	1.236956	1.219430	1.235546	0.015459	1.25

Note: All three absorption experiments were conducted independently under identical operating conditions. SD denotes the sample standard deviation (n = 3), and RSD was calculated as 100 × SD/mean.

Table S5. Tentative assignment of characteristic ^{13}C NMR signals in time-series and phase-separated DETA/DMSO/H_2O samples**.**

The assignments were made based on previously reported DETA–CO_2 ^{13}C NMR studies and related amine–CO_2 systems. Literature chemical shifts were used as references rather than as the sole criteria for assignment because differences in solvent composition, hydrogen-bonding interactions, ionic environments, and proton exchange may cause slight chemical shift deviations.

Sample	Experimental δ (ppm)	Literature δ (ppm)	Tentative assignment	Confidence level	Reference
Fresh	50.96	50.65	DETA methylene carbon (C2-related)	High	[33]
Fresh	40.31	39.98	DETA methylene carbon (C1-related)	High	[33]
Fresh	39.14	39.39 (D_2O); 40.59 (DETA/DMSO)	DMSO methyl carbon	High (component)	[16,34]
10 min	164.40	~164.5 (Fig. 4)	B5-related terminal mono-carbamate carbonyl (tentative)	Medium–high	[33]
10 min	50.58	50.65 (free DETA)	Shifted DETA methylene environment; individual protonation state unresolved	Medium	[33]
10 min	40.00; 39.08	39.98 (DETA); 39.39 / 40.59 (DMSO)	DETA-related / DMSO-overlapping aliphatic signals; individual assignments unresolved	Low–medium	[16,33,34]
20 min	164.41	~164.5 (Fig. 4)	B5-related carbamate carbonyl (tentative)	Medium–high	[33]
20 min	164.37	~164.5 (B type); 164.44 (D type)	Overlapping B5/D3-related carbamate carbonyl environments	Medium	[22,33]
20 min	163.92	163.95 (C type)	C3-related central mono-carbamate carbonyl (tentative)	Medium	[33]
20 min	50.03; 49.84	50.65 (free DETA); 37–52 (DETA region)	A2/B2-related DETA methylene environments; site-specific assignment tentative	Low–medium	[33]
20 min	48.65; 48.58	48.12 (C type); 48.39 (D type)	B3/D2-related methylene environments; overlapping possibilities	Low–medium	[33]
20 min	39.76; 39.02	39.98; 39.40 (DETA); 39.39 / 40.59 (DMSO)	DETA-related / DMSO-overlapping signals; not uniquely resolved	Low	[16,33,34]
30 min	164.30	~164.5 (B type); 164.44 (D type)	B5/D3-related carbamate carbonyl; overlapping signals	Medium	[22,33]
30 min	164.26 (shoulder)	~164.5 (B type); 164.44 (D type)	Possible unresolved B5/D3-related carbonyl contribution	Low–medium	[22,33]
30 min	163.80	163.95 (C type)	C3-related carbamate carbonyl (tentative)	Medium	[33]
30 min	49.43; 49.31; 48.65; 48.61	37–52 (DETA region); 48.12; 48.39	DETA methylene signals; different protonation/carbamate environments, not uniquely assigned	Low–medium	[33]
60 min	164.22	~164.5 (B type); 164.44 (D type)	B5/D3-related carbamate carbonyl (tentative)	Medium	[22,33]
60 min	163.73	163.95 (C type); 163.68 (carbamate)	C3/D3-related carbamate carbonyl; individual structure unresolved	Low–medium	[14,33]
60 min	48.45; 48.22; 48.07	48.12 (C type); 48.39 (D type)	DETA methylene carbon environments; assignment not unique	Medium	[33]
60 min	39.05 (weak)	39.40 (DETA); 39.39 / 40.59 (DMSO)	Unresolved DMSO-overlapping / E-type-related aliphatic signal	Low	[16,33,34]
150 min	164.20	~164.5 (Fig. 4)	B5-related terminal mono-carbamate carbonyl (tentative)	Medium	[33]

Sample	Experimental δ (ppm)	Literature δ (ppm)	Tentative assignment	Confidence level	Reference
150 min	163.83	163.95 (C type); 164.44 (D type)	D3-related carbamate carbonyl (tentative); overlap with other environments possible	Low–medium	[33]
150 min	163.78	164.40; 164.01 (E-type carbonyls)	E6-related dicarbamate carbonyl (tentative); exact site unresolved	Low–medium	[33]
150 min	48.29	48.39 (D type)	D2-related DETA methylene environment (tentative)	Medium	[33]
150 min	45.62; 45.53	37–52 (DETA region); no unique match	Overlapping C2/A2-related DETA methylene environments (tentative)	Low	[33,41]
Rich phase	164.05	164.01 (E type); 164.12 (carbamate)	CO_2-derived carbamate carbonyl; B/C/D/E candidates not uniquely resolved	Medium (class)	[14,33]
Rich phase	164.00	164.01 (E type); 164.12 (carbamate)	CO_2-derived carbamate carbonyl; candidate structures unresolved	Medium (class)	[14,33]
Rich phase	163.61	163.68 (DETA carbamate); 163.95 (C type)	CO_2-derived carbamate carbonyl; specific B/C/D assignment uncertain	Medium	[14,33]
Rich phase	160.45 (weak)	160.71	Possible HCO_3^-/CO_3^{2-}-related carbon signal (tentative)	Low–medium	[33]
Rich phase	48.13	48.39 (D-type CH_2); 48.12 (C-type CH_2)	D2-related DETA methylene environment (tentative)	Medium	[33]
Rich phase	45.26	37–52 (DETA carbon region); no unique match	A2-related DETA methylene environment (tentative)	Low–medium	[33,41]
Rich phase	38.69	39.39 (D_2O); 40.59 (DETA/DMSO)	DMSO methyl carbon	High (component)	[16,34]
Lean phase	164.29–163.88 (unresolved region)	163–165 (carbamate region)	Multiple overlapping CO_2-derived carbamate-related carbonyl signals; no unique structures	Low–medium	[33,41]
Lean phase	159.99 (weak)	160.71	Possible HCO_3^-/CO_3^{2-}-related carbon signal; assignment not unique	Low	[33]
Lean phase	39.05	39.39 (D_2O); 40.59 (DETA/DMSO)	DMSO methyl carbon; potential overlap with other aliphatic signals	Medium (component)	[16,34]

Notes. Literature δ values for DETA-related signals are from Ref. [33] (Hartono et al., 2007) and Ref. [14] (Zhang et al., 2024), with the broad carbamate region supported by Ref. [41] (Ye et al., 2017). For DMSO methyl carbon, 39.39 ppm was reported in D_2O by Gottlieb et al. (Ref. [34]), whereas 40.59 ppm was reported for a DETA/DMSO solid–liquid absorbent in Ref. [16] (Wang et al., 2023; NMR samples prepared using deuterated methanol). These literature values were measured under different conditions and serve only as supporting comparisons for the DMSO-related signals in the present DETA/DMSO/H_2O system; they are not exact expected positions for every sample. For the overlapping 39–40 ppm features observed at 10, 20 and 60 min, a contribution from DMSO is possible but a unique assignment is not established. Approximately 164.5 ppm for the B-type carbonyl was estimated from Figure 4 of Ref. [33], not quoted as an exact tabulated value. Ranges such as 37–52 ppm and 163–165 ppm denote broad spectral regions rather than site-specific peak matches. The structural nomenclature in Ref. [33] is related to, but does not necessarily use identical carbon numbering to, Figure 3a of the present manuscript. Overlapping resonances, fast proton exchange, and differences in solvent composition limit unique assignments for some signals. Assignment confidence was evaluated qualitatively based on agreement with reported chemical shifts, spectral resolution and overlap, consistency with the experimental chemical environment, and the uniqueness of the proposed assignment. High confidence was assigned mainly to characteristic signals of known absorbent components; medium confidence indicated assignments supported by relevant spectral evidence but not uniquely confirmed; and low confidence indicated weak, overlapping, or insufficiently resolved signals. The designation “Medium (class)” indicates confidence in the general chemical environment rather than in a specific molecular isomer. These levels represent qualitative assessments of assignment reliability, not statistically determined confidence intervals.

Table S6. Effect of D3(BJ) dispersion correction on the relative electronic energies of candidate carbamate structures and ion-pair models.

**Structural pair**	**B3LYP ΔE_{rel} (kJ mol^{-1})**	**B3LYP-D3(BJ) ΔE_{rel} (kJ mol^{-1})**	**Lower-energy structure after D3(BJ)**
B^{-} / C^{-}	20.26	12.43	B^{-}
D^{2-} / E^{2-}	19.16	7.78	D^{2-}
$B^{-}\cdots DETAH^{+}$ / $C^{-}\cdots DETAH^{+}$	14.19	-0.91	C-type
$[D^{2-}\cdots DETAH^{+}]^{-}$ / $[E^{2-}\cdots DETAH^{+}]^{-}$	18.61	1.58	D-type

Note. For each structural pair, ΔE_{rel} = E(second-listed structure) − E(first-listed structure); positive values favor the first-listed structure, whereas negative values favor the second-listed structure. The uncorrected B3LYP values are those reported in Table S1 for SMD(H_2O). The D3(BJ) values were independently calculated from the final single-point electronic energies of the eight Gaussian 16 outputs at B3LYP-D3(BJ)/6-311++G(d,p) with SMD(H_2O), using the original B3LYP-optimized geometries (1 hartree = 2625.499638 kJ mol^{-1}).

These values are relative electronic energies (ΔE_{rel}), not Gibbs free-energy differences (ΔG). The ordering of the isolated B^{-}/C^{-} and D^{2-}/E^{2-} pairs remains unchanged. The B/C ion-pair ordering reverses slightly after D3(BJ) correction (−0.91 kJ mol^{-1}), while the D-type ion pair remains lower in electronic energy by 1.58 kJ mol^{-1}. The small ion-pair energy differences should not be interpreted as reliable predictions of solution-phase species populations.

Cartesian Coordinates of DFT-Optimized Structures

The Cartesian coordinates of the optimized structures used in the DFT analysis are provided below. The structures correspond to the B−, C−, D²−, and E²− carbamate models and their representative ion-pair structures.

Computational level: B3LYP/6-311++G(d,p). Solvent model: SMD(water) (as used for the original optimization calculations).

B⁻

Charge = -1; Multiplicity = 1

Atom	X (Å)	Y (Å)	Z (Å)
N	2.957267	1.802555	-0.128812
H	1.962923	1.925515	0.046030
H	3.412557	1.964107	0.767256
C	3.192456	0.408270	-0.534721
H	2.713378	0.253090	-1.504978
H	4.265603	0.273951	-0.696277
C	2.728952	-0.664208	0.453959
H	3.219903	-0.487445	1.416100
H	3.076959	-1.646061	0.090128
N	1.276357	-0.656784	0.684983
H	1.092387	-1.142932	1.557822
C	0.516915	-1.340774	-0.371012
H	0.759579	-0.881702	-1.332992
H	0.791891	-2.404675	-0.445516
C	-0.984178	-1.250361	-0.136770
H	-1.493465	-1.855671	-0.889417
H	-1.226026	-1.686364	0.842870
N	-1.447370	0.132138	-0.231146
H	-0.781253	0.818488	0.096923
C	-2.774862	0.476943	-0.000809
O	-3.020616	1.709768	0.216075
O	-3.651123	-0.441628	-0.051950

C⁻

Charge = -1; Multiplicity = 1

Atom	X (Å)	Y (Å)	Z (Å)
N	-2.992429	-0.594661	1.029403
H	-2.158417	-0.841483	1.557227
C	-2.584640	-0.108174	-0.297530
H	-2.134942	-0.940503	-0.843847
H	-3.485372	0.180453	-0.848135
C	-1.632242	1.091184	-0.309508
H	-2.090894	1.916323	0.235043
H	-1.504891	1.418544	-1.348920
N	-0.305173	0.846102	0.265590
C	0.722362	0.282638	-0.619379
H	0.269321	0.172721	-1.608615
H	1.544439	0.998505	-0.726363
C	1.335954	-1.061489	-0.221889
H	2.202486	-1.208226	-0.883495
H	1.721643	-1.006956	0.796997

N	0.383737	-2.182588	-0.293690
C	0.070265	1.436684	1.471064
O	1.302411	1.387515	1.795479
O	-0.830417	1.987264	2.186910
H	0.874537	-3.046870	-0.081841
H	-3.414389	0.176442	1.542431
H	0.047854	-2.274618	-1.249653

D^{2-}

Charge = -2; Multiplicity = 1

Atom	X (Å)	Y (Å)	Z (Å)
N	-3.125688	0.406580	-0.280534
H	-2.735624	-0.514349	-0.139712
C	-2.203796	1.409725	-0.805071
H	-1.735178	0.994411	-1.700595
H	-2.785496	2.274065	-1.128256
C	-1.128888	1.890702	0.174327
H	-1.613936	2.207280	1.100959
H	-0.650719	2.783740	-0.262003
N	-0.129415	0.864539	0.512248
H	0.329003	1.153491	1.372215
C	0.916030	0.728049	-0.512805
H	0.457787	0.388508	-1.445667
H	1.395444	1.697027	-0.726265
C	2.006588	-0.249098	-0.100166
H	2.796630	-0.221528	-0.852018
H	2.453916	0.087806	0.846395
N	1.505099	-1.617114	0.008146
H	0.586136	-1.701722	0.419941
C	2.352902	-2.680450	0.306651
O	1.804127	-3.760061	0.706424
O	3.600754	-2.520897	0.131208
C	-4.135732	0.723124	0.625948
O	-4.653481	-0.250691	1.267387
O	-4.505079	1.933908	0.725759

E^{2-}

Charge = -2; Multiplicity = 1

Atom	X (Å)	Y (Å)	Z (Å)
N	-2.697514	0.039162	0.440574
H	-1.999738	-0.432281	1.001176
C	-2.193300	0.594886	-0.812417
H	-1.718298	-0.214475	-1.370720
H	-3.043139	0.934739	-1.406709
C	-1.214715	1.766820	-0.662937
H	-1.695265	2.567801	-0.104241
H	-0.997098	2.150797	-1.666744
N	0.054004	1.447851	0.001414
C	1.155212	0.941349	-0.832359
H	0.799233	0.928180	-1.865214
H	1.996130	1.640996	-0.788299

C	1.683861	-0.449420	-0.483935
H	2.576022	-0.615017	-1.095234
H	2.007155	-0.462521	0.562528
N	0.724017	-1.526262	-0.790895
H	-0.094369	-1.421027	-0.196371
C	0.334164	1.971588	1.265632
O	-0.617702	2.497777	1.928514
O	1.533600	1.886770	1.689158
C	-3.653910	0.702425	1.212145
O	-3.751638	0.344521	2.431574
O	-4.389874	1.570855	0.650149
H	1.139535	-2.415662	-0.526435

B^{-}···$DETAH^{+}$

Charge = 0; Multiplicity = 1

Atom	X (Å)	Y (Å)	Z (Å)
N	-4.724444	2.070660	-1.828377
H	-4.078573	2.493718	-2.489695
H	-4.590149	1.064332	-1.891503
C	-4.440983	2.527294	-0.463715
H	-4.512930	3.616053	-0.442787
H	-5.224719	2.153266	0.199519
C	-3.118701	2.064685	0.144510
H	-3.057182	0.974930	0.126436
H	-3.032295	2.399091	1.179721
N	-1.899349	2.563069	-0.577300
H	-1.065934	1.995269	-0.234823
C	-1.606543	4.022875	-0.388958
H	-2.485497	4.590196	-0.693464
H	-1.444683	4.167268	0.679990
C	-0.382719	4.475876	-1.168638
H	-0.189866	5.506425	-0.864889
H	0.486607	3.882816	-0.859416
N	-0.600629	4.462426	-2.625227
H	0.147996	4.984872	-3.071074
H	-0.515908	3.512417	-2.978801
H	-2.004560	2.357706	-1.574118
N	3.426877	-4.999435	1.014913
H	2.720386	-4.383923	0.619678
H	3.976209	-5.326751	0.222838
C	4.295892	-4.208533	1.899822
H	3.698719	-3.879365	2.754206
H	5.069034	-4.869184	2.301756
C	4.994789	-3.008589	1.256195
H	5.589143	-3.364037	0.408710
H	5.700929	-2.584046	1.990122
N	4.062436	-1.988912	0.751110
H	4.562057	-1.407809	0.084029
C	3.541903	-1.106524	1.804481
H	3.054323	-1.718346	2.567897
H	4.349609	-0.551308	2.306799
C	2.542640	-0.098353	1.256224
H	2.255878	0.583585	2.058876

H	3.025458	0.506612	0.476320
N	1.350163	-0.767608	0.740454
H	1.513883	-1.679111	0.334945
C	0.295072	-0.073943	0.178710
O	-0.548780	-0.729673	-0.506716
O	0.221518	1.182715	0.412198

C^{-}···$DETAH^{+}$

Charge = 0; Multiplicity = 1

Atom	X (Å)	Y (Å)	Z (Å)
N	-3.214351	-0.448162	-0.249222
H	-2.596580	-0.766162	0.494654
C	-2.400734	-0.136432	-1.433899
H	-1.935996	-1.061928	-1.780874
H	-3.069094	0.196657	-2.233892
C	-1.331954	0.946748	-1.252907
H	-1.816246	1.877653	-0.958050
H	-0.850789	1.122039	-2.222039
N	-0.285839	0.646013	-0.267227
C	0.904378	-0.091418	-0.723511
H	0.798494	-0.235146	-1.800990
H	1.793840	0.525453	-0.562859
C	1.151007	-1.450591	-0.070578
H	2.131964	-1.793867	-0.412145
H	1.217552	-1.324082	1.015247
N	0.159099	-2.469317	-0.460659
H	-0.752727	-2.202145	-0.098371
C	-0.260568	1.307398	0.952053
O	0.795551	1.166458	1.669896
O	-1.258516	2.007085	1.305023
H	0.391153	-3.342295	0.005898
H	-3.632178	0.412472	0.097841
N	-0.117447	2.417231	6.942018
H	-0.577429	3.167743	6.432795
H	-0.669537	1.577469	6.784409
C	1.245944	2.217847	6.439200
H	1.795854	3.153894	6.550575
H	1.749930	1.486397	7.075605
C	1.371026	1.691889	5.011062
H	0.852181	0.736041	4.914153
H	2.420280	1.544569	4.749225
N	0.785004	2.592642	3.964284
H	0.789287	2.063438	3.035393
C	1.464055	3.921428	3.810913
H	1.263055	4.505734	4.708843
H	2.535459	3.726768	3.744751
C	0.987024	4.662411	2.570350
H	1.503507	5.624107	2.565869
H	1.312575	4.118549	1.675504
N	-0.465311	4.903179	2.587783
H	-0.679970	5.658879	1.944246
H	-0.937795	4.078658	2.218293
H	-0.202197	2.754401	4.180297

$[D^{2-}\cdots DETAH^{+}]^{-}$

Charge = -1; Multiplicity = 1

Atom	X (Å)	Y (Å)	Z (Å)
N	-3.243498	1.468443	-0.144855
H	-2.971915	0.500728	-0.045604
C	-2.279822	2.321726	-0.833356
H	-2.024500	1.836253	-1.778410
H	-2.771126	3.262189	-1.087028
C	-1.004879	2.642656	-0.047447
H	-1.283992	3.082500	0.913155
H	-0.451199	3.414422	-0.608387
N	-0.160542	1.468618	0.223276
H	0.478293	1.715623	0.974224
C	0.655527	1.066332	-0.930991
H	-0.006698	0.776239	-1.751154
H	1.273921	1.900677	-1.298698
C	1.584603	-0.091582	-0.597826
H	2.228438	-0.280329	-1.458527
H	2.239034	0.197272	0.236521
N	0.837349	-1.307474	-0.283211
H	-0.041570	-1.164264	0.194380
C	1.461888	-2.501135	0.025020
O	0.765262	-3.411537	0.570559
O	2.697165	-2.624286	-0.288937
C	-4.029412	1.928465	0.910224
O	-4.563359	1.039742	1.653543
O	-4.199627	3.179931	1.040512
N	3.822946	-7.993193	-0.005775
H	3.378205	-7.772399	0.881424
H	3.099513	-7.928938	-0.718036
C	4.894767	-7.035966	-0.297791
H	5.626426	-7.078497	0.510989
H	5.415101	-7.359205	-1.202539
C	4.460556	-5.594132	-0.549795
H	3.746264	-5.554203	-1.374681
H	5.320337	-4.972345	-0.804881
N	3.784744	-4.943512	0.620855
H	3.347820	-4.031405	0.285013
C	4.671404	-4.672519	1.799917
H	5.023425	-5.628941	2.185775
H	5.523866	-4.103553	1.426472
C	3.953441	-3.886498	2.886043
H	4.689448	-3.702524	3.670879
H	3.654347	-2.908861	2.489695
N	2.827962	-4.633216	3.472711
H	2.570601	-4.196905	4.353125
H	2.010167	-4.541506	2.873998
H	3.001796	-5.531554	0.919103

[$E^{2-}\cdots$DETAH^{+}]$^{-}$

Charge = -1; Multiplicity = 1

Atom	X (Å)	Y (Å)	Z (Å)
N	0.396803	3.123451	-2.101739
H	1.143432	3.614962	-1.629837
C	-0.953643	3.574254	-1.775241
H	-0.953178	4.664597	-1.826472
H	-1.639072	3.219785	-2.547967
C	-1.495628	3.112606	-0.415679
H	-1.524336	2.025498	-0.392642
H	-2.529619	3.469075	-0.335672
N	-0.747704	3.572354	0.758826
C	-1.107633	4.881659	1.326911
H	-1.932434	5.277619	0.729813
H	-1.481592	4.743822	2.346514
C	0.004769	5.927956	1.373020
H	-0.373128	6.772155	1.957383
H	0.862275	5.520365	1.919202
N	0.374808	6.437113	0.039221
H	0.776162	5.678695	-0.506389
C	-0.047094	2.652819	1.545906
O	0.155997	1.485318	1.079109
O	0.358452	3.042336	2.689123
C	0.699308	1.798377	-2.371584
O	1.916394	1.446723	-2.299104
O	-0.258000	1.022939	-2.716999
H	1.121984	7.118072	0.147494
N	0.493592	-2.685405	1.123198
H	1.397402	-2.658530	0.658383
H	0.359789	-1.771821	1.551126
C	-0.575421	-2.941899	0.152124
H	-0.347764	-3.869310	-0.376187
H	-1.508495	-3.115554	0.693853
C	-0.866267	-1.816490	-0.837424
H	-1.123324	-0.901202	-0.301009
H	-1.697825	-2.082479	-1.492124
N	0.293479	-1.466564	-1.722922
H	0.075970	-0.548956	-2.205817
C	0.676256	-2.511535	-2.728188
H	1.052745	-3.376276	-2.181966
H	-0.233876	-2.792532	-3.259603
C	1.720552	-1.992602	-3.705406
H	1.965018	-2.822784	-4.370630
H	1.274976	-1.204360	-4.324591
N	2.947977	-1.551055	-3.023959
H	3.706015	-1.522276	-3.699022
H	2.819434	-0.590802	-2.705429
H	1.107297	-1.265966	-1.134515
